# Impact of Bone Marrow Aspirate Tregs on the Response Rate of Younger Newly Diagnosed Acute Myeloid Leukemia Patients

**DOI:** 10.1155/2018/9325261

**Published:** 2018-07-04

**Authors:** Mario Delia, Paola Carluccio, Anna Mestice, Claudia Brunetti, Francesco Albano, Giorgina Specchia

**Affiliations:** Hematology and Bone Marrow Transplantation Unit, Department of Emergency and Organ Transplantation, University of Bari, Bari, Italy

## Abstract

Acute myeloid leukemia (AML) is widely considered a distinct clinical entity with a well-defined molecular and genetics-based prognosis. Particularly in a younger patient, the therapeutic approach depends largely on diagnostic risk stratification, which has an impact on the outcome after therapy. We added Treg evaluation to the usual molecular and cytogenetics profile in the AML younger patients' diagnostic bone marrow aspirate (dBMA) in order to search for any correlation between Tregs and overall response (OR) as well as survival (OS) rates. We studied 23 AML young patients, all treated with standard induction chemotherapy: OR (complete remission (CR) + CR incomplete (CRi)) was documented in 10 of 23 patients (44%); there were two partial responder patients. The optimal dBMA Treg cut-off value for predicting response to treatment (≥21/*μ*L) was obtained by ROC curve analysis. However, in multivariate analysis, apart from the expected impact of the molecular/cytogenetic risk (*p* = 0.049) and NPM mutation (*p* = 0.001), dBMA Tregs ≥ 21/*μ*L was not correlated with OR. Actually, higher dBMA Tregs were associated with the good intermediate molecular/cytogenetic risk group (*p* = 0.02), whose median OS was confirmed to be better as compared with that of the poor risk group (18 versus 5 months, *p* = 0.05) and equal to the dBMA Tregs ≥ 21/*μ*L group (5 versus 5 months, *p* = 0.902), respectively. The possible prognostic value of such an immunological player as BMA Tregs in the diagnostic and successive phases of AML needs to be confirmed in larger patient numbers.

## 1. Introduction

Considerable progress has been made in understanding the pathogenesis of acute myeloid leukemia (AML) [1] and in the development of diagnostic assays [2], including European Leukemia Net (ELN) 2017 updated therapies [3]. But although AML subgroups have distinct prognoses and different therapeutic needs [2], the therapeutic approach remains based on induction chemotherapy followed by allogeneic stem cell transplantation (allo-HSCT) in the case of poor prognosis AML subgroups [4]. In this scenario, the contribution of an immunological player such as T regulatory cells (Tregs), evaluated in diagnostic bone marrow aspirate (dBMA), might suggest novel insights that could be useful in terms of prognosis and outcome.

In fact, while in solid tumors, the role of Tregs seems to be associated with tumor escape from immunosurveillance and, consequently, a worse outcome [5, 6], in AML; its action is still not fully understood [7]. Actually, in selected lymphomas, higher Tregs seem to be associated with a better outcome [8], while they have shown conflicting results in terms of a worse [9, 10] or better prognosis [11] for AML patients. In fact, in allo-HSCT, the ability of Tregs to suppress the function of other T cells and accordingly, to limit the immune response, to regulate immune homeostasis, and to maintain self-tolerance, has recently been reviewed [12] and correlated with beneficial effects on the outcome of AML patients after allo-HSCT [13]. Here, we have applied our previous experience regarding the effects of Treg graft contents on immunological recovery [14] to dBMA results obtained in AML patients. In particular, the aim of our preliminary study was to investigate the role of AML diagnostic phase-Tregs in terms of a possible prognostic impact on the overall response (OR) and outcome.

## 2. Materials and Methods

### 2.1. Patients

We prospectively analyzed newly diagnosed AML patients (<65 years) treated at our institution between March 2016 and March 2018. Patients gave written informed consent to the collection of personal data in accordance with the Declaration of Helsinki and Italian laws. The study included 23 AML patients (13 males and 10 females, median age 55 years, range 20–65). Fluorescence in situ hybridization for molecular rearrangements was performed on bone marrow samples, as previously reported [15]. According to cytogenetic-molecular risk stratification [16], 3 (13%) patients were assigned to the favorable, 12 the intermediate (52%), and 8 (35%) the adverse prognosis group. Molecular evaluation (i.e., NPM, FLT3, and CEBPA) was performed in all cases: NPM1 (A or B mutation) and FLT3 mutations (ITD or D835) were positive in 6 (26%) and 5 (22%) patients, respectively. There were no CEBPA-positive cases. Median values of white blood cells (WBC) were 18430/*μ*L and of dBMA Tregs 21/*μ*L. The Treg study population, together with the B and NK cell distribution in BMA, is summarized in Table 1. All patients underwent induction chemotherapy (i.e., “3 + 7”) with cytarabine 100 mg/mq, intravenously, on days 1 to 7 and an anthracycline [daunorubicin 60 mg/mq on days 1 to 3 or mitoxantrone 10 mg/mq on days 1 to 3] and thereafter intermediate dose cytarabine for consolidation (up to 2 cycles) [3] or at higher doses (FLAG-Ida for all patients) as a bridge to allotransplantation [17, 18] for nonresponder patients. AML response was evaluated according to the ELN 2017 [3].

### 2.2. Flow Cytometry

To determine the percentage and the absolute count of CD3 and CD4 T cell subsets, 50 *μ*L of whole marrow blood was stained with CD45 PerCP-Cy™5.5, CD3 FITC, CD4 PE-Cy7™, CD8 APC-Cy7, CD16 and CD56 PE, and CD19 APC monoclonal antibodies (MoAbs) (BD Multitest 6-color TBNK) in a calibrated number of fluorescent beads (Trucount, BD Pharmingen). For Treg identification, 100 *μ*L of marrow blood was incubated with a lyophilised pellet of CD45RA FITC, CD25 PE, CD127 PerCP-Cy 5.5, HLA-DR PE-CY™7, CD39 APC, and CD4 APC-H7 MoAbs (BD Pharmingen). Samples were processed according to the manufacturer's guidelines and acquired on a DB FACSCanto II Flow Cytometer. The absolute number (cells/*μ*L) of positive cells was calculated by comparing cellular events to bead events using BD FACSCanto clinical software (version 3).

### 2.3. Treg Populations

BMA Tregs we found were the following (Table 1):

- CD4+/CD127^low^/CD25^high^
- CD4+/CD45RA-/CD25^high^/CD127^low^
- CD4+/CD45RA-/CD127^low^/CD25^high^/DR+/39+

**Figure 1:**
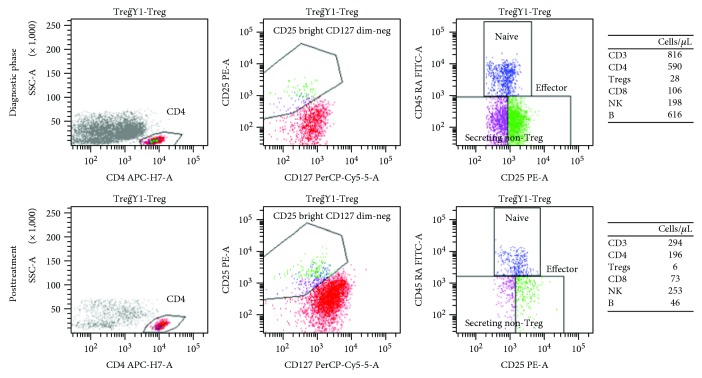
Flow cytometry plot in a responder patient: diagnostic and posttreatment evaluation (the T/B/NK population is summarized in the grid).

The population we studied in our analysis was the CD4+/CD45RA-/CD25^high^/CD127^low^ group (Figure 1).

### 2.4. Statistical Analysis

The Mann–Whitney rank sum test was used to compare absolute cell counts while chi-square or Fisher's exact test (2-tailed) was performed to compare proportions. The comparison of the dBMA population with the posttreatment group was performed with the paired *t*-test or Wilcoxon signed-rank test as appropriate. The variables analyzed for a correlation with OR were age, WBC, and integrated molecular-cytogenetic risk; the NPM mutation; the FLT3 ITD or D835 mutation; NPM^mut^FLT3^wt^Normal Karyotype; and dBMA Tregs and de novo versus secondary AML. The variables analyzed for the correlation with Tregs were integrated molecular-cytogenetic risk; the NPM mutation; the FLT3 ITD or D835 mutation; and de novo versus secondary AML and WBC. Covariates in the multivariate logistic regression models were chosen by stepwise-with-backward elimination variable selection procedures. The discriminatory power of the dBMA Tregs value to predict response was assessed by estimating the area under the ROC curve (AUC). The optimal cut-off was determined by maximizing both sensitivity and specificity, computed at the optimal cut-off, as reported along with the 95% confidence intervals. Overall survival (OS) curves were plotted with the Kaplan-Meier method and compared by log-rank test, censoring patients (6 out of 23) at allotransplantation.

The significance was defined as a *p* value of <0.05.

## 3. Results

OR (complete remission (CR) + CR incomplete (CRi)) was documented in 10 of 23 patients (44%). There were two partial responder patients. The variables impacting on OR are reported in Table 2.

**Figure 2:**
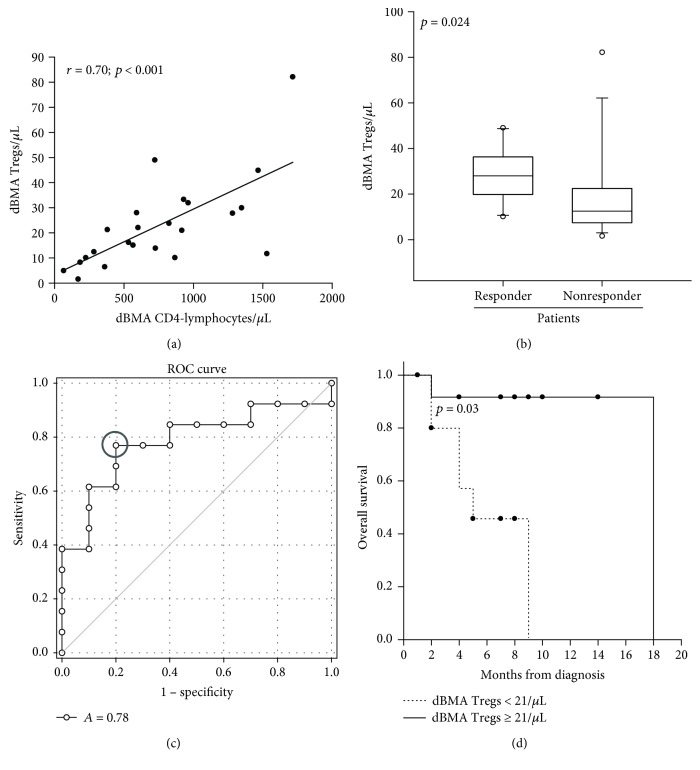
(a) Diagnostic bone marrow aspirate (dBMA) CD4-lymphocyte correlation with Tregs (*r* = 0.70, *p* < 0.001). (b) Median dBMA Treg value in responder and nonresponder patients (28 versus 13/*μ*L, Mann–Whitney rank sum test, *p* = 0.024). (c) ROC curve: AUC analysis (AUC, 0.80; 95% CI, 0.5902–0.9791, *p* = 0.02), 21/*μ*L, optimal dBMA Treg cut-off value for predicting response to treatment, yielding 77% sensitivity (95% CI, 53% to 92%) and 80% specificity (95% CI, 44% to 97%). (d) Median OS in ≥21/*μ*L and <21/*μ*L dBMA Tregs (18 versus 5 months, log-rank test, *p* = 0.03).

Figure 2 shows a dBMA CD4-lymphocyte correlation with dBMA Tregs (*r* = 0.7, *p* < 0.001), the association between OR and higher dBMA Tregs (*p* = 0.024), the optimal dBMA Tregs cut-off value for predicting response to treatment (≥21/*μ*L, AUC = 0.78, *p* = 0.02), and accordingly, OS patient stratification according to dBMA Tregs (*p* = 0.03).

The BMA population modifications after treatment (i.e., at day 28 after the start of “3 + 7”) are summarized in Table 3.

The dBMA Treg ≥ 21/*μ*L correlation with integrated molecular-cytogenetic risk; the NPM mutation; the FLT3 ITD or D835 mutation; and de novo versus secondary AML and WBC are summarized in Table 4: the molecular-cytogenetic risk association (*p* = 0.020) was demonstrated.

**Figure 3:**
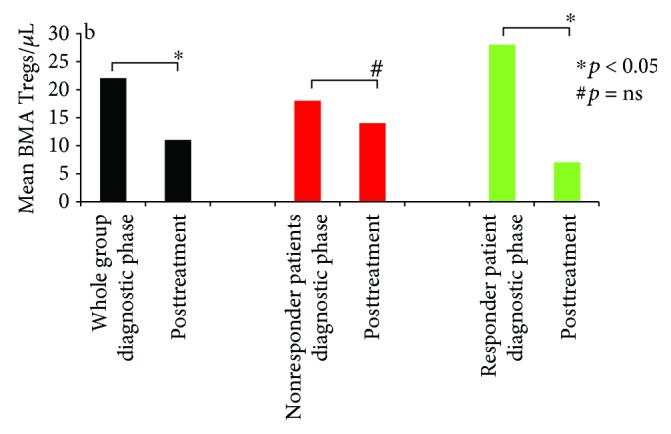
After treatment, mean dBMA Tregs in the whole (black), responder (green), and nonresponder (red) patient groups, respectively (22 versus 12/*μ*L, paired *t*-test, ^∗^*p* = 0.038, 28 versus 7/*μ*L, Wilcoxon signed-rank test ^∗^*p* = 0.030; 18 versus 14/*μ*L, paired *t*-test, ^#^*p* = 0.32).

The responder and nonresponder patients' mean dBMA Tregs after treatment are reported in Figure 3.

**Figure 4:**
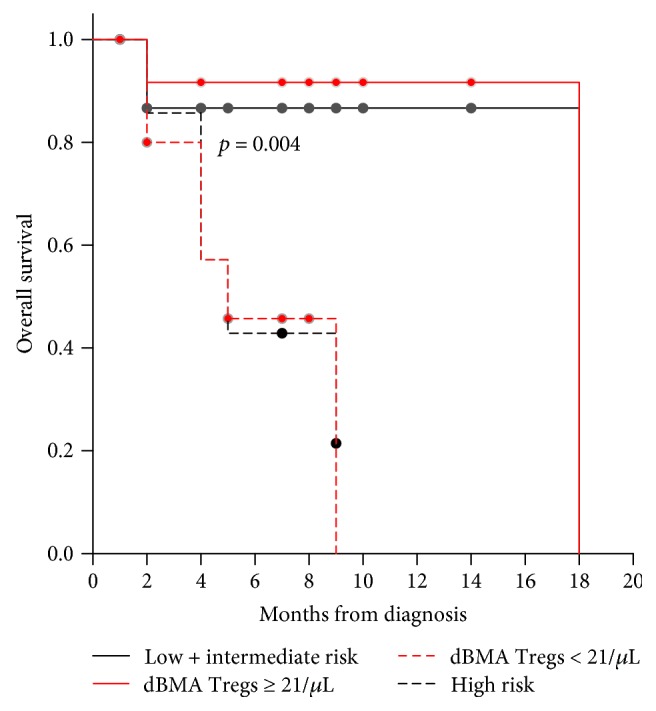
Median OS patients in Tregs < 21/*μ*L; Tregs ≥ 21/*μ*L; poor and low-intermediate risk group, respectively (5, 18, 5, and 18 months; log-rank test; *p* = 0.004).

The whole group median OS was 18 months. OS according to Tregs and molecular-cytogenetic risk is reported in Figure 4 (median OS in dBMA Tregs ≥ 21/*μ*L and the low-intermediate risk group, 18 versus 18 months, *p* = 0.902; median OS in the high and low-intermediate risk-group, 5 versus 18 months, *p* = 0.05, all pairwise multiple comparison procedures, Holm-Sidak method).

## 4. Discussion

The possible role of Tregs in terms of their effects on the prognosis in the AML setting has been amply hypothesized [9–11] but is still not clearly understood. The key point in the present investigation was to restrict the analysis to as homogeneous a patient's group as possible, limiting any pre- and posttreatment confounding factors. Hence, we analyzed dBMA Tregs from younger newly diagnosed AML patients, all treated with the “3 + 7” regimen according to a sequential enrollment procedure.

Primarily, we were interested in evaluating whether our study population was similar to the one reported in the literature in regard to the higher Treg frequencies in dBMA that we chose to analyze. In fact, it is well known that Tregs increase not only in the peripheral blood [9, 10, 19–21] but also in the bone marrow, where they seem to be higher and also more immunosuppressive [10, 19]. Of note, studying the same population CD4+/CD25^high^/CD127^low^, Shenghui et al. [10] reported frequencies of 11.8 (% of CD4+ T cells); our relative dBMA frequencies were similar: 10 (median value, 56/*μ*L, Table 1).

Given the possible expansion [22, 23] and bearing in mind the immunosuppressive weight of dBMA Tregs (CD4+/CD45RA-/CD25^high^/CD127^low^) which finally favor AML cells [7, 19], we investigated the possible correlation of dBMA Tregs with the outcome and obtained the ROC curve optimal cut-off value to predict a better OR (≥21/*μ*L, Figure 2(c)) and OS (Figure 2(d)). Consequently, at univariate analysis, dBMA Tregs (as both continuous (Figure 2(b)) and categorical variables (Table 2) were correlated with OR as well as NPM mutation, molecular-cytogenetic risk, and *de novo* AML (Table 2). However, while the impact of Tregs on OR was not confirmed at multivariate analysis (Table 2) but molecular-cytogenetic risk remained, we found a correlation between Tregs and the factors known to have an impact on AML outcome [1] (Table 4).

Therefore, our data might appear conflicting with the observations showing diagnostic Tregs to be correlated with poor prognosis in both the peripheral blood [9, 10] and the bone marrow [10], while on the contrary, in our study population, the higher number of Tregs benefit was due to their association with the good-intermediate molecular-cytogenetics risk group and not to the Treg value itself (Table 2, Figure 4). Of note, Szczepanski et al. [9] did not demonstrate any correlation between Treg levels and the cytogenetic subgroup, although their study population was the peripheral one and the prognostic stratification referred to karyotypic analysis [24] and not to the integrated molecular-cytogenetics stratification we used [3, 16]. Of course, the reason why, in our study population, dBMA Tregs were higher in AML with a better prognosis risk remains to be elucidated and study of a larger patient cohort will be needed to confirm or dismiss the association.

All the same, in the peripheral blood, it has been already shown [20] that treatment-induced lymphopenia is not a random process and susceptibility to intensive chemotherapy differs between T cells subsets (i.e., CD4, CD8, and Tregs). In fact, although in the bone marrow we confirmed that CD4+ and CD8+ T cell levels did not differ after treatment, a statistically significant difference was documented for Tregs (Table 3). Of note, in responder patients, Tregs statistically decreased (Figure 3), confirming the correlation with OR and suggesting their possible utility to monitor response after treatment. In fact, albeit in peripheral blood Tregs, a secondary response to inflammation caused by induction chemotherapy and to cytokine secretion has already been reported to justify the expansion of Tregs after treatment in CR patients [9]. Obviously, in this regard, a longer monitoring time might clarify the correlation between the Treg levels and the risk of AML relapse.

On the other hand, a stereotyped immunologic response to chemotherapy in patients with AML that is independent of the AML etiology, cytogenetics, or molecular characteristics was also proposed [21] to understand the increase of peripheral Tregs during treatment. Analyzing BMA Tregs, we did not find this (Figure 3). Moreover, in the early phases after induction (i.e., day 17), another group [11] supposed that Tregs might also foster the recovery of normal hematopoietic cells, thus promoting higher CR rates and a better OS.

## 5. Conclusions

It is widely recognized [7] that BMA Tregs are higher in, and act at, the neoplastic AML site (i.e., bone marrow) primarily favoring leukemia growth but, taken together, our data, while confirming this point, might suggest that their final effect is probably due to both disease-associated and chemotherapy-induced modifications. Moreover, several studies [9, 10, 19–21] suggest a prognostic impact of Tregs in AML patients receiving intensive chemotherapy, but Tregs persistence after treatment [11, 21] and in CR patients [9] might even sustain opposite mechanisms such as recognition of leukemia-associated antigens (as supposed in the allo-setting) [25] on the one hand and leukemia-relapse promotion on the other. In this regard, sequential measurements (i.e., diagnostic, day 14, and recovery phase, after obtaining CR and during consolidation) of Tregs in a larger number of patients are needed to confirm the prognostic value of BMA Tregs in the diagnostic phase of AML and to monitor their value after treatment and to clarify any correlation with leukemia relapse.

## Figures and Tables

**Table 1:** Bone marrow aspirate T/NK/B cell distribution.

	Median value/*μ*L	Range
CD3+	1350	4587–504
CD4+	721	64–1715
*Tregs CD4+/CD127^low^/CD25^high^*	56	6–201
**Tregs CD4+/CD45RA-/CD127**^**low**^**/CD25**^**high**^^∗^	**21**	**2–82**
*Tregs CD4+/CD45RA-/CD127^low^/CD25^high^/DR+/39+*	*5*	1–27
CD8+	370	56–1338
CD16+/56+	225	90–1536
CD19+	265	0–2560

CD16+/CD56+: NK; CD19+: B cells. ^∗^Study population.

**Table 2:** Factors affecting overall response.

	Response		*p*
	Yes	No	
	*n* = 10	*n* = 13	
*Age*			
Years, median value	52	56	ns
*WBC*			
WBC/μL, median value	9565	18430	ns
*Molecular/cytogenetic group^@^*, *n(%)*			**0.027** ^**b**^ **; 0.049** ^**d**^
Poor	1 (10)	7 (54)	
Intermediate	6 (60)	6 (46)	
Good	3 (30)	0 (0)	
*NPM/FLT3*, *n(%)*			
NPM^mut^	6 (60)	0 (0)	**0.002** ^**c**^ **; 0.001** ^**d**^
NPM^wt^	4 (40)	13 (100)	
FLT3 ITD+ or D835+	1 (10)	4 (31)	ns
FLT3^wt^	9 (90)	9 (69)	
NPM^mut^/FLT3^wt^/NK	3 (30)	0 (0)	ns
No (NPM^mut^/FLT3^wt^/Nk)	7 (70)	13 (100)	
*dBMA Tregs^#^*, *n(%)*			**0.036** ^**c**^; ns^d^
<21/μL	2 (20)	9 (69)	
≥21/μL	8 (80)	4 (31)	
*De novo AML*, *n(%)*			**0.046** ^**c**^; ns^d^
Yes	10 (100)	8 (61)	
No	0 (0)	5 (39)	

^@^According to ELN 2010 [ITD allelic ratio not performed]. ^b^Chi-square test. ^c^Fisher exact test. ^d^Multivariate stepwise-backward elimination procedure. Bold values are statistically significant (*p* < 0.05). WBC: white blood cells; dBMA Tregs: diagnostic bone marrow aspirate T regulatory cells; NK: normal karyotype; AML: acute myeloid leukemia.

**Table 3:** Bone marrow aspirate T, NK, and B population in diagnosis and after treatment phase.

	Diagnosis	After treatment	*p*
CD3+/*μ*L, *mean value (mv)*	1350	960	0.125^a^
CD4+/*μ*L, *mv*	721	481	0.116^a^
CD8+/*μ*L, *mv*	370	373	0.870^a^
Tregs/*μ*L, *mv*	21	12	**0.038** ^**a**^
CD16+/56+, *median value (med v)*	225	256	0.742^b^
CD19+/*μ*L, *med v*	265	12	**0.008** ^**b**^

^a^Paired *t*-test. ^b^Wilcoxon signed-rank test. CD16+/56+: NK; CD19+: B cells. Bold values are statistically significant (*p* < 0.05).

**Table 4:** Correlation between dBMA Tregs and AML-related prognostic factors.

	Tregs < 21/*μ*L	Tregs ≥ 21/*μ*L	*p*
	*n* = 11	*n* = 12	
*Molecular/cytogenetic group^@^*, *n(%)*			**0.020** ^b^
Poor	7 (64)	1 (8)	
Intermediate	3 (27)	9 (75)	
Good	1 (9)	2 (17)	
*NPM/FLT3*, *n(%)*			
NPM^mut^	2 (18)	4 (33)	0.64^c^
NPM^wt^	9 (82)	8 (67)	
FLT3 ITD+ or D835+	3 (27)	2 (17)	0.64^c^
FLT3^wt^	8 (73)	10 (83)	
*WBC count*			0.99^c^
<100 × 10e3/*μ*L	10 (91)	10 (83)	
≥100 × 10e3/*μ*L	1(9)	2 (17)	
*De novo AML*, *n(%)*			0.155^c^
Yes	7 (64)	11 (92)	
No	4 (36)	1 (8)	

^@^According to ELN 2010 [16] [ITD allelic ratio not performed]. ^b^Chi-square test. ^c^Fisher exact test. Bold value is statistically significant (*p* < 0.05).

## Data Availability

The data used to support the findings of this study are available from the corresponding author upon request.
